# Painless Method of Excising the Whole Tongue

**Published:** 1879-06

**Authors:** 


### PAINLESS METHOD OF EXCISING THE WHOLE TONGUE.

---

Mr. Richard Barwell, Surgeon to, and Lecturer on Surgery at, Charing Cross Hospital, London, makes (*Lancet*, April 19, 1879) the following remarks on a case of a man on whom he performed excision of the whole tongue nine days ago.

The disease was a large epithelioma situated as far back in the organ as the anterior pillar of the fauces, occupying chiefly the left side; that is to say, the tumor itself and the ulceration were confined to that side, yet the condition called icthyosis extended across and some distance on the right of the raphe. Now my late colleague, Mr. Fairlie Clarke, pointed out some years ago, (*Med.-Chir. Trans.*, vol. lvii., p. 155) that this morbid state is the immediate precusor, or, indeed, the first stage of epithelioma. To take away a part of the tongue and to leave behind an ichthyotic portion would be a grave mistake. It was necessary, therefore, in this case to remove the whole breadth of the organ from a point very near the epiglottis. I desire to fix your attention upon the method I adopted on account of its ease both to surgeon and patient, and the absence of bleeding or external mutilation; especially as you will find in works on surgery, much used by students and practioners, certain methods of operation described and figured of which I entirely disapprove. For instance, Regnoli's operation consists in cutting away the whole floor of the mouth by incisions along the middle line and round the body of the lower jaw, then dragging the tongue through the opening down upon the front of the neck, and severing it from its base.

Another method is to slit soft parts and jaw from the mouth to the hoyid bone, and by dragging the parts asunder to lay bare the root of the tongue. These operations cause much hemorrhage, are very dangerous, and produce horrible mutilation. I have no hesitation in saying that they should only be mentioned as I mention them now—namely, as relics of a past, and, in this particular region, of a barbarous stage of surgery. Even the division of muscles, etc., passing between the jaw, hyoid bone, and the tongue, as suggested by Sir James Paget, so as to enable the surgeon to drag the last named part out of the mouth, is quite unnecessary, because, as you have seen, the tongue can be removed *e situ* with the greatest ease as far back, if necessary, as the epiglottis; nay, if it were desirable, that valve could, as far as the mere mechanism of the operation is concerned, be removed with the tongue from the hyoid bone.

The method itself is very simple. The instruments required are a small scalpel, one or two Liston's needles, and an ecraseur, or better, two ecraseurs. When the patient is well under the influence of the anæsthetic, place a gag between the jaws, draw the tongue a little forward, and pass through the raphe a string, with which the organ is to be simply controlled, not dragged out of the mouth, which must be avoided. An incision, about a quarter or a third of an inch long, is now made from the hyoid bone forward, and strictly in the middle line. Thus far you will see my operation resembles Nunneley's, except that my incision is further back and shorter; but from this point the methods differ, for that surgeon passed by means of a seton-needle the loop of an ecraseur chain into the floor of the mouth through the frenum of the tongue, and then dragged the part to be removed forward through the loop; and, although he could remove considerable parts by these means, he could hardly get at the whole organ, and I think his opening into the mouth too short and direct, nor did he eliminate pain.

By my method, when the raphe of the mylo-hyoid has been divided, the knife is laid aside, the genio-hyoid and genio-hyoglossus muscles are seperated from their fellows by the handle of the scalpel or by the finger if the surgeon have a small finger-tip, and the root of the tongue is readily reached; but the mouth is not to be opened here. An armed Liston's needle is now placed in the wound, and the forefinger of the other hand between the diseased side of the tongue and the jaw, as far back, as it will go, viz., a little beyond the last molar tooth—and to this point the needle is guided, taking care to keep it rather nearer to the bone, than to the side of the tongue; here it pierces the mucous membrane, enters the mouth, and the thread, being released, is withdrawn, a loop of cord being left behind. The same thing is then done for the other side, except that here a loop in the mouth is unnecessary. The ecraseur is now taken in hand; it must have one end of the wire detached and bent into a sort of hook at as sharp an angle as the material will bear. Tie an end of the last placed thread in the bend of this hook; then by traction on the other end, that in the mouth, draw the wire along the track of the needle. When the metal appears in the mouth just beyond the last molar tooth, pull the wire gently through till the nozzle of the ecraseur is close to the supra-hyoid wound; then detach the thread and pass the wire hook into the loop of twine that enters the mouth on the diseased side of the tongue, and by gentle traction draw the metal from thus far back in the mouth, out at the hyoid wound, and attach it to the body of the instrument. Before screwing the wire tight, pass a finger along the dorsum of the tongue and ascertain its exact position. I am not afraid of its lying too far forward—it might easily, without care, sit too far back, also it might slip away from the desired place as the screw is used; therefore, having fixed the exact line along which the tongue is to be severed, I place my finger where that line intersects the raphe on the dorsum of the tongue; to it I pass the Liston's needle, letting its point project a line or two, and taking care that the wire lies behind it; by this means the ecraseur can be guided exactly along the required plane. When the base of the tongue has been cut through, and the wire has come out at the wound, the loop of the same or of another ecraseur is passed over the tip of the tognue into the line of incision, and the tissues, small in quantity but very vascular, which attach the tongue to the floor of the mouth, slowly cut through, when the whole organ is severed, and is removed from between the lips.

Now to recall your attention to tne man himself. He lost during the operation not more than ten drops of blood, and none since. He has in front of the hyoid bone a very small scar of an already healed wound, and no other external mutilation. He has lost the whole of the tongue, well clear of the disease, as you see by the specimen, and within a line or two of the epiglottis; yet he has no fever, his temperature is normal, and he takes tepid liquids without difficulty. Whenever I have asked him if he is in or has suffered any pain, he invariably answered in the negative. It seems strange, at first sight, that an organ so sensitive as the tongue can be removed without the production of a moment's pain, especially as a good deal of suffering follows the usual modes of excision; yet, when we have considered the matter together, you will see that this is a necessary result of my method of operation. By avoiding any dragging of the tongue forward, but, on the contrary, getting the ecraseur wire around it *in situ*, and by keeping that wire, just previous to its entrance into the mouth, rather near though not close to the ramus of the jaw, I divide the sensory nerve of the tongue, the lingual-gustatory, close to the bone; it then retracts into its groove, and the whole wound must of necessity, be insensible to pain. Therefore the man could immediately after the operation take abundance of liquid nourishment, avoided fever, and the part has rapidly healed. I would suggest, though I have not yet had an opportunity of reducing the proposal to practice, that when a less portion of the tongue has to be removed the lingual-gustatory nerve of one or both sides, according to the extent of amputation, might with advantage be divided on the ramus of the jaw.—*Abstract of Medical Science.*

